# Sensitivity Analysis Using a Reduced Finite Element Model for Structural Damage Identification

**DOI:** 10.3390/ma14195514

**Published:** 2021-09-23

**Authors:** Qiuwei Yang, Xi Peng

**Affiliations:** 1 School of Civil and Transportation Engineering, Ningbo University of Technology, Ningbo 315211, China; s7ilent@sina.com; 2 Engineering Research Center of Industrial Construction in Civil Engineering of Zhejiang, Ningbo University of Technology, Ningbo 315211, China

**Keywords:** sensitivity analysis, reduced model, damage identification, eigenvalue, eigenvector, feedback-generalized inverse

## Abstract

Sensitivity analysis is widely used in engineering fields, such as structural damage identification, model correction, and vibration control. In general, the existing sensitivity calculation formulas are derived from the complete finite element model, which requires a large amount of calculation for large-scale structures. In view of this, a fast sensitivity analysis algorithm based on the reduced finite element model is proposed in this paper. The basic idea of the proposed sensitivity analysis algorithm is to use a model reduction technique to avoid the complex calculation required in solving eigenvalues and eigenvectors by the complete model. Compared with the existing sensitivity calculation formulas, the proposed approach may increase efficiency, with a small loss of accuracy of sensitivity analysis. Using the fast sensitivity analysis, the linear equations for structural damage identification can be established to solve the desired elemental damage parameters. Moreover, a feedback-generalized inverse algorithm is proposed in this work in order to improve the calculation accuracy of damage identification. The core principle of this feedback operation is to reduce the number of unknowns, step by step, according to the generalized inverse solution. Numerical and experimental examples show that the fast sensitivity analysis based on the reduced model can obtain almost the same results as those obtained by the complete model for low eigenvalues and eigenvectors. The feedback-generalized inverse algorithm can effectively overcome the ill-posed problem of the linear equations and obtain accurate results of damage identification under data noise interference. The proposed method may be a very promising tool for sensitivity analysis and damage identification based on the reduced finite element model.

## 1. Introduction

The derivatives of the eigenvalue and eigenvector with respect to the system parameters are usually referred to as the sensitivity coefficients [1,2,3]. Sensitivity analysis is often used in the following areas: finite element model (FEM) updating [4,5], structural optimization design [6,7], vibration control [8,9], damage identification [10,11], and so on. Therefore, there has been much interest in efficient computation methods for sensitivity analysis. The classical sensitivity analysis approaches are the modal superposition method [12] and Nelson’s method [13]. The key idea of the mode superposition method is to express the eigenvector sensitivity as a linear combination of all modes. This leads to great limitations in the application of mode superposition method, since only a few modes are available for large-scale structures. In view of this, several improved modal superposition algorithms [14,15,16,17] are proposed by using only the partial lower modes. Using these methods, sensitivity analysis for the damping systems [18,19] and complex-valued systems [20,21] are studied by some researchers. Generally, the calculation formula of this kind of method is complex, especially for the higher-order sensitivity analysis. Relatively speaking, Nelson’s method has an obvious advantage that only the eigenvector of interest is required. Using the inverse iteration technique, Lin et al. [22,23] further improved the computation efficiency of Nelson’s method. Adhikari and Friswell [24] extended Nelson’s method for sensitivity analysis of nonviscously damped systems. Wu et al. [25] improved Nelson’s method for sensitivity analysis with distinct and repeated eigenvalues by reducing the condition number of the coefficient matrix. Using Nelson’s method, Guedria et al. [26] performed the second-order sensitivity analysis for symmetric and asymmetric damped systems with distinct eigenvalues. Wang and Dai [27,28] further studied the eigensensitivities for symmetric and asymmetric damped systems with repeated eigenvalues. Ruiz et al. [29] presented a general framework for sensitivity analysis when tracking specific mode shapes selected beforehand. Lin and Ng [30] used Nelson’s method to analyze the eigenpair sensitivities of fractional vibration systems. Recently, Yang and Peng [31] proposed an exact method for calculating the eigenvector sensitivities, which can be seen as an improvement of Nelson’s method due to its simple operation.

However, the above sensitivity calculation formulas are generally derived from the complete FEM of the structure, which require a large amount of calculation for large-scale structures. On the other hand, only a few degrees of freedom (DOFs) of the structure can be measured in engineering practice due to the limitation of modal measurement technology. As a result, the reduced FEM [32,33,34,35] is often used in structural mechanics analysis. It is very necessary to study the fast sensitivity analysis approach based on the reduced FEM. To this end, a new sensitivity analysis algorithm is proposed in this paper by using the reduced FEM. Subsequently, structural damage identification based on the proposed sensitivity analysis is studied. A feedback-generalized inverse algorithm is also proposed for solving the damage identification equations more accurately.

The presentation of this work is organized as follows. In Section 2, the common formulas for calculating the eigenvalue and eigenvector sensitivities are briefly reviewed. In Section 3, the new algorithm for fast sensitivity analysis is proposed by using the IRS-z reduced model. The application of this algorithm in structural damage identification is illustrated in Section 4. Numerical and experimental examples are presented in Section 5 to verify the proposed method. Finally, the conclusions of this work are summarized in Section 6.

## 2. Review of Sensitivity Calculation Formulas

In this section, the common formulas for calculating the eigenvalue and eigenvector sensitivities are briefly reviewed. Assuming that K and M denote the n×n stiffness and mass matrices of structural FEM, the free vibration modes of the structure can be obtained by solving the following generalized eigenvalue equation [12,13,14,15,16,17,18,19,20,21] as:

(1) $$(K-\lambda_{j}M)\varphi_{j}=0$$

(2) $$\varphi_{j}^{T}M\varphi_{j}=1$$

where $\lambda_{j}$ and $\varphi_{j}$ denote the *j*th eigenvalue and eigenvector, j=1,2,⋯,n. Clearly, K,M,$\lambda_{j}$, and $\varphi_{j}$ are the functions of the physical parameters $p_{i}$, such as elastic modulus, cross-sectional area, and density. By taking a partial derivative of Equation (1) with respect to $p_{i}$, one has:

(3) $$(K-\lambda_{j}M)\frac{\partial \varphi_{j}}{\partial p_{i}}=(\frac{\partial \lambda_{j}}{\partial p_{i}}M+\lambda_{j}\frac{\partial M}{\partial p_{i}}-\frac{\partial K}{\partial p_{i}})\varphi_{j}$$

where $\frac{\partial \lambda_{j}}{\partial p_{i}}$ and $\frac{\partial \varphi_{j}}{\partial p_{i}}$ are the first-order eigenvalue and eigenvector sensitivities, respectively. From Equation (1), one can obtain:

(4) $$\varphi_{j}^{T}(K-\lambda_{j}M)=0$$

Using Equations (4) and (2), the eigenvalue sensitivity can be obtained by multiplying Equation (3) by $\varphi_{j}^{T}$ as:

(5) $$\frac{\partial \lambda_{j}}{\partial p_{i}}=\varphi_{j}^{T}\frac{\partial K}{\partial p_{i}}\varphi_{j}-\lambda_{j}\varphi_{j}^{T}\frac{\partial M}{\partial p_{i}}\varphi_{j}$$

For eigenvector sensitivity, Fox and Kapoor [12] proposed the calculation formula based on the modal superposition as:

(6) $$\frac{\partial \varphi_{j}}{\partial p_{i}}=\sum_{r=1,r\neq j}^{n}c_{jr}\varphi_{r}-c_{jj}\varphi_{j}$$

where:

(7) $$c_{jr}=\frac{\varphi_{r}^{T}(\frac{\partial K}{\partial p_{i}}-\lambda_{j}\frac{\partial M}{\partial p_{i}})\varphi_{j}}{\lambda_{j}-\lambda_{r}}$$

(8) r≠j

(9) $$c_{jj}=\frac{1}{2}\varphi_{j}^{T}\frac{\partial M}{\partial p_{i}}\varphi_{j}$$

It is known that Equation (6) is inefficient, since all the eigenvectors are needed in computation. In contrast, Nelson’s method [13] has the great advantage that only the eigenvector of interest is required. Its basic idea is to convert the eigenvector sensitivity into the sum of the general solution and the special solution. Then, the general solution and the special solution are solved, respectively. Based on Nelson’s method, Yang and Peng [31] present a direct calculation formula for eigenvector sensitivity as:

(10) $$\frac{\partial \varphi_{j}}{\partial p_{i}}=\Theta^{-1}\Pi \varphi_{j}$$

(11) $$\Pi =\frac{\partial \lambda_{j}}{\partial p_{i}}M+\lambda_{j}\frac{\partial M}{\partial p_{i}}-\frac{\partial K}{\partial p_{i}}-c_{jj}\lambda_{j}I$$

(12) $$\Theta =K-\lambda_{j}M+\lambda_{j}\varphi_{j}\varphi_{j}^{T}M$$

Note that it is still necessary to further improve the computational efficiency of Equation (10) for large-scale structures. For a structure with more than thousands of DOFs, the computational burden of Equation (10) mainly lies in the solving process of eigenvalue $\lambda_{j}$ and eigenvector $\varphi_{j}$ from Equation (1). Therefore, it is very meaningful to study a fast calculation method that can reduce the above calculation burden. To this end, a new sensitivity analysis method based on reduced FEM is proposed in the next section.

## 3. The Proposed Algorithm for Fast Sensitivity Calculation

As stated before, the existing sensitivity calculation formulas are generally derived from the complete FEM of the structure, which leads to the large amount of calculation. On the other hand, only a few measurement points can be arranged on the structure in engineering practice due to the limitation of modal measurement technology. In view of this, a new algorithm for fast sensitivity analysis is proposed in this work by using the reduced FEM.

Model reduction is a commonly used algorithm to fast estimate some low eigenvalues and eigenvectors of structures by reducing the order of the original structural model to a smaller one. Guyan method [32,33] is the earliest method for FEM reduction. By partitioning the DOFs into the master DOF and slave DOF, Equation (1) can be rewritten as:

(13) $$\left[\begin{array}{cc} K_{mm} & K_{ms}\\ K_{sm} & K_{ss} \end{array}\right]\left\{\begin{array}{c} \varphi_{j}^{m}\\ \varphi_{j}^{s} \end{array}\right\}=\lambda_{j}\left[\begin{array}{cc} M_{mm} & M_{ms}\\ M_{sm} & M_{ss} \end{array}\right]\left\{\begin{array}{c} \varphi_{j}^{m}\\ \varphi_{j}^{s} \end{array}\right\}$$

where the subscripts “m” and “s” represent the number of the master and slave DOFs, m+s=n. Note that the master DOFs are associated with the measured DOFs in structural dynamic testing. From Equation (13), the Guyan reduced model can be derived as [12,13]:

(14) $$K_{r}\varphi_{j}^{m}=\lambda_{j}M_{r}\varphi_{j}^{m}$$

(15) $$K_{r}=T_{0}^{T}KT_{0}$$

(16) $$M_{r}=T_{0}^{T}MT_{0}$$

(17) $$T_{0}=\left[\begin{array}{c} I\\ -K_{ss}^{-1}K_{sm} \end{array}\right]$$

(18) $$\varphi_{j}=T_{0}\varphi_{j}^{m}$$

where $K_{r}$ and $M_{r}$ are the reduced stiffness and mass matrices, and $T_{0}$ is the DOF transformation matrix. It is clear that the dimensions of $K_{r}$ and $M_{r}$ are both m×m, which just match with the measured DOFs. By solving Equation (14), $\lambda_{j}$ and $\varphi_{j}^{m}$ can be obtained as the approximate solutions of the low eigenvalues and eigenvectors of the complete FEM. Clearly, the calculation time of solving Equation (14) is far less than that of solving Equation (1) due to m<<n. Based on Guyan reduction, the improved reduced system (IRS) reduced model is proposed in [34] by considering the first-order inertia item to improve the computation accuracy as:

(19) $$K_{1r}\varphi_{j}^{m}=\lambda_{j}M_{1r}\varphi_{j}^{m}$$

(20) $$K_{1r}=T_{1}^{T}KT_{1}$$

(21) $$M_{1r}=T_{1}^{T}MT_{1}$$

(22) $$T_{1}=T_{0}+SMT_{0}M_{r}^{-1}K_{r},\;$$

(23) $$S=\left[\begin{array}{cc} 0 & 0\\ 0 & K_{ss}^{-1} \end{array}\right]$$

Furthermore, the IRS-2 reduced model is proposed in [35] by considering the first two inertia items as:

(24) $$K_{2r}\varphi_{j}^{m}=\lambda_{j}M_{2r}\varphi_{j}^{m}$$

(25) $$K_{2r}=T_{2}^{T}KT_{2},$$

(26) $$M_{2r}=T_{2}^{T}MT_{2}$$

(27) $$T_{2}=T_{0}+SMT_{0}M_{r}^{-1}K_{r}+{\left(SM\right)}^{2}T_{0}{\left(M_{r}^{-1}K_{r}\right)}^{2}$$

As an extension for the IRS reduced method, IRS-z reduced model is proposed in this paper by considering the first z inertia items as follows:

(28) $$K_{zr}\varphi_{zj}^{m}=\lambda_{zj}M_{zr}\varphi_{zj}^{m}$$

(29) $$K_{zr}=T_{z}^{T}KT_{z}$$

(30) $$M_{zr}=T_{z}^{T}MT_{z}$$

(31) $$T_{z}=\sum_{q=0}^{z}{\left(SM\right)}^{q}T_{0}{\left(M_{r}^{-1}K_{r}\right)}^{q}$$

Using the IRS-z reduced model, the fast sensitivity calculation formulas can be derived as follows. Using $\varphi_{j}=T_{z}\varphi_{zj}^{m}$, Equations (5) and (10) can be rewritten as:

(32) $$\frac{\partial \lambda_{j}}{\partial p_{i}}={\left(T_{z}\varphi_{zj}^{m}\right)}^{T}\frac{\partial K}{\partial p_{i}}(T_{z}\varphi_{zj}^{m})-\lambda_{j}{\left(T_{z}\varphi_{zj}^{m}\right)}^{T}\frac{\partial M}{\partial p_{i}}(T_{z}\varphi_{zj}^{m})$$

(33) $$\frac{\partial \varphi_{j}}{\partial \alpha_{i}}={\left[K-\lambda_{zj}M+\lambda_{zj}T_{z}\varphi_{zj}^{m}{\left(T_{z}\varphi_{zj}^{m}\right)}^{T}M\right]}^{-1}\Pi (T_{z}\varphi_{zj}^{m})$$

Equations (32) and (33) are the proposed formulas for quickly computing the sensitivities of eigenvalues and eigenvectors. As will be shown in the numerical examples, the proposed formulas may increase efficiency, with a small loss of accuracy of sensitivity analysis compared with the existing sensitivity calculation formulas.

Finally, it is necessary to discuss how to determine the suitable value of z in the IRS-z reduced model. Note that the inertias in Equation (31) are approximated by Guyan reduced FEM. It is known that the eigenvalues calculated by the Guyan reduced model are generally greater than the exact values. As a result, the accuracy of the IRS-z reduced model first increases, and then decreases with increasing z. Therefore, a criterion to determine the suitable value of z is needed. In this work, the sum of squares of the calculated eigenvalues for each reduced FEM with different z is used as the criterion. That is:

(34) $$\Theta_{z}=\lambda_{z1}^{2}+\lambda_{z2}^{2}+\cdots +\lambda_{zm}^{2}$$

The reduced FEM with a minimum value of $\Theta_{z}$ is associated with the most suitable value of z.

## 4. Application in Structural Damage Identification

The fast sensitivity analysis method proposed above can be used in many engineering fields, such as structural damage identification, model updating, vibration control, etc. In this section, the application of the above sensitivity analysis in damage identification is illustrated as follows.

Generally, structural damage only leads to the reduction in its stiffness matrix. Correspondingly, the stiffness matrix change can be expressed as the sum of the elemental stiffness matrix multiplied by a damage parameter as:

(35) $$\Delta K=\sum_{i=1}^{N}x_{i}K_{i}$$

where N represents the total number of elements in FEM, $K_{i}$ denotes the i-th elemental stiffness matrix, and $x_{i}$ denotes the corresponding damage parameter ($0\leq x_{i}\leq 1$). In particular, $x_{i}=0$ represents that the i-th element is not damaged and $x_{i}=1$ denotes that the i-th element is completely damaged. Using Taylor series expansion, the changes of eigenvalue and eigenvector before and after damage can be approximated as:

(36) $$\Delta \lambda_{j}=\sum_{i=1}^{N}x_{i}\frac{\partial \lambda_{j}}{\partial \alpha_{i}}$$

(37) $$\Delta \varphi_{j}=\sum_{i=1}^{N}x_{i}\frac{\partial \varphi_{j}}{\partial \alpha_{i}}$$

where $\frac{\partial \lambda_{j}}{\partial \alpha_{i}}$ and $\frac{\partial \varphi_{j}}{\partial \alpha_{i}}$ can be computed by the fast sensitivity analysis algorithm proposed above. Combining Equation (36) with (37), the linear equations of damage identification can be obtained as:

(38) A⋅x=b

(39) $$x={\left(x_{1},x_{2},\cdots ,x_{N}\right)}^{T}$$

(40) $$b=\left\{\begin{array}{c} \Delta \lambda_{j}\\ \Delta \phi_{j}^{m} \end{array}\right\}$$

(41) $$A=\left[\begin{array}{cccc} \frac{\partial \lambda_{j}}{\partial \alpha_{1}} & \frac{\partial \lambda_{j}}{\partial \alpha_{2}} & \cdots & \frac{\partial \lambda_{j}}{\partial \alpha_{N}}\\ \frac{\partial \phi_{j}^{m}}{\partial \alpha_{1}} & \frac{\partial \phi_{j}^{m}}{\partial \alpha_{2}} & \cdots & \frac{\partial \phi_{j}^{m}}{\partial \alpha_{N}} \end{array}\right]$$

Commonly, the generalized inverse is used to solve Equation (38) to obtain structural damage parameters as:

(42) $$x_{gi}=A^{+}\cdot b$$

where $x_{gi}$ is the generalized inverse (GI) solution, $A^{+}$ is the Moore–Penrose generalized inverse [35,36]. For damage identification equations, the condition number of the coefficient matrix A may be very large. For this reason, the calculation results obtained by Equation (42) may be seriously distorted when the data used in the calculation contain random noise [37,38]. In view of this, this paper proposes a feedback-generalized inverse to compute damage parameters more accurately. To this end, a diagonal matrix with small parameters is firstly added to the coefficient matrix of Equation (38) as:

(43) $$\bar{A}\cdot x=b$$

(44) $$\bar{A}=A+\left[\varepsilon \right]$$

(45) $$\left[\varepsilon \right]=\left[\begin{array}{cccc} \varepsilon_{11} & 0 & \cdots & 0\\ 0 & \ddots & \cdots & 0\\ \vdots & \vdots & \varepsilon_{ii} & \vdots \\ 0 & 0 & \ldots & \ddots \end{array}\right]$$

The purpose of the above operation is to improve the ill condition of the linear equations. The diagonal elements $\varepsilon_{ii}$ in the matrix [ε] are calculated by:

(46) $$\varepsilon_{ii}=\gamma \times a_{\max}\times 2^{\left(\frac{a_{ii}}{a_{\max}}-1\right)}$$

(47) $$\gamma =\{\begin{array}{c} 0.05\;\;if\;\;cond(A)\geq 100\\ 0\;\;\;if\;\;cond(A)<100 \end{array}$$

where $a_{\max}$ is the maximum value of all diagonal elements of A, and cond(A) denotes the condition number of A. From Equation (43), the first solution of x can be obtained as:

(48) $$x_{gi1}={\bar{A}}^{+}\cdot b$$

According to $x_{gi1}$, less than 0.05 of the calculated values of damage parameters can be determined to relate with the undamaged elements. From this feedback, Equation (43) can be further reduced by removing the related coefficients associated with undamaged elements as:

(49) $${\bar{A}}^{*}\cdot {\bar{x}}^{*}=b$$

where ${\bar{x}}^{*}$ is the reduced damage parameter vector, and ${\bar{A}}^{*}$ is the corresponding coefficient matrix. From Equation (49), the feedback-generalized inverse (FGI) solution of x can be obtained as:

(50) $$x_{fgi}={\left({\bar{A}}^{*}\right)}^{+}\cdot b$$

**Figure 1:**
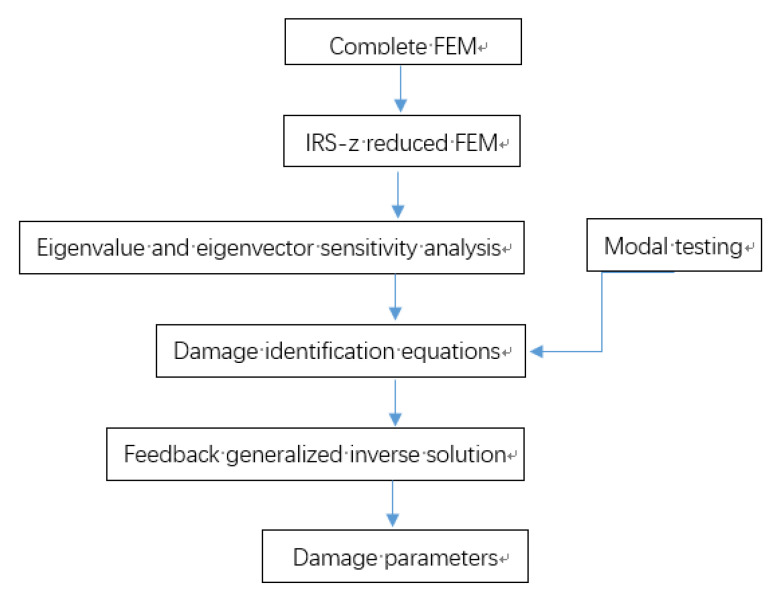
Flowchart of the proposed method.

Finally, structural damages are evaluated according to $x_{fgi}$. This feedback process can be repeated until satisfactory results are obtained. In summary, a flowchart, as shown in Figure 1, is given to illustrate the whole technique more clearly.

## 5. Numerical and Experimental Examples

### 5.1. Numerical Example

**Figure 2:**
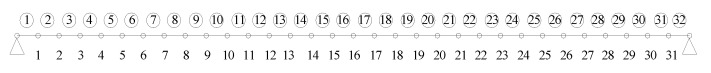
A beam structure.

**Figure 3:**
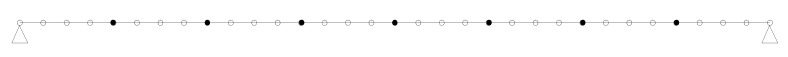
Locations of the master DOFs (denoted by the black circle points).

The proposed method is verified firstly by the beam structure, as shown in Figure 2. The basic physical parameters of the structure are: the cross-sectional area $A=1.8\times 10^{-4}$ m^2^, the elastic modulus E=71 GPa, the density $\rho =2.2\times 10^{3}$ Kg/m^3^, the length of each element L=0.1 m, and the moment of inertia $I=5.4\times 10^{-10}$ m^4^. The structure has 64 DOFs (31 translational DOFs and 33 rotational DOFs). The translational DOFs of nodes 4, 8, 12, 16, 20, 24, and 28 are chosen as the master DOFs for constructing the reduced FEM, as shown by the black circle points in Figure 3.

**Figure 4:**
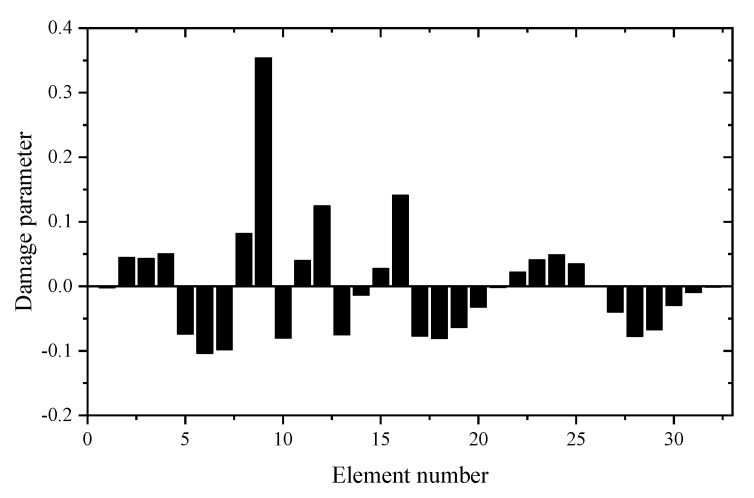
The calculation results using generalized inverse without feedback (element 9 is damaged).

**Figure 5:**
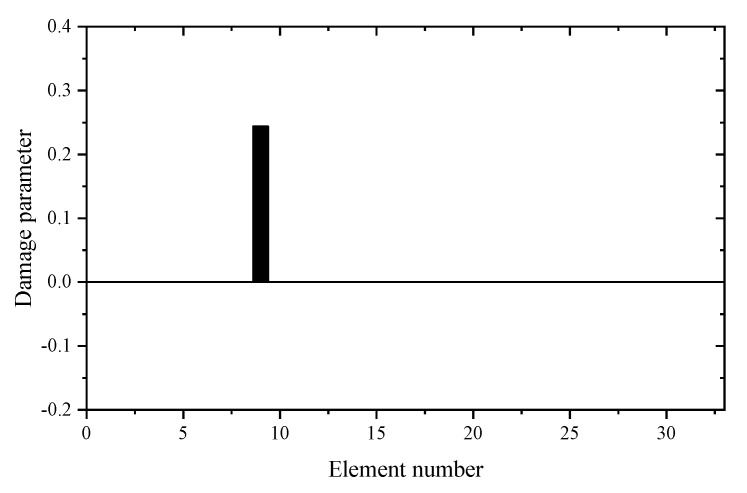
The calculation results using generalized inverse after feedback (element 9 is damaged).

Using the reduced model, Table 1 gives the sum of squares of the first seven eigenvalues calculated by different z. For comparison, the calculation results using the complete model are also given in Table 1. It can be seen from Table 1 that the $\Theta_{z}$ with *z* = 3 is the smallest. This means that the reduced model with *z* = 3 is the best condensation model and should be used in the following sensitivity calculation. Table 2 and Table 3 present the calculation results of frequency and eigenvector sensitivities based on the complete and IRS-3 reduced model, respectively. It can be seen from Table 2 that the first five eigenvalue sensitivities obtained by the reduced model are very close to those obtained by the complete model. From Table 3, the eigenvector sensitivity obtained by the reduced model is exactly the same as that obtained by the complete model. The computation times by the complete and reduced models are 0.125 and 0.109 s, respectively. These results show that the proposed sensitivity calculation formulas based on the reduced FEM are reasonable and effective. Next, the damage identification method is verified by assuming that the elastic modulus of element 9 in the structure is reduced to 80%. Moreover, 5% random noise is added to the data to simulate the measurement error. Using the first two eigenpair sensitivities, the first calculation result calculated by the generalized inverse is given in Figure 4. It can be found from Figure 4 that unit 9 is the most likely damage unit position. Subsequently, Figure 5 presents the final calculation result based on the feedback. One can see from Figure 5 that unit 9 is the only damage unit, and the calculated damage parameters are very close to the assumed value (0.2). It is clear that the calculated results obtained by feedback have a higher accuracy than the calculated results without feedback.

### 5.2. Experimental Example

**Figure 6:**
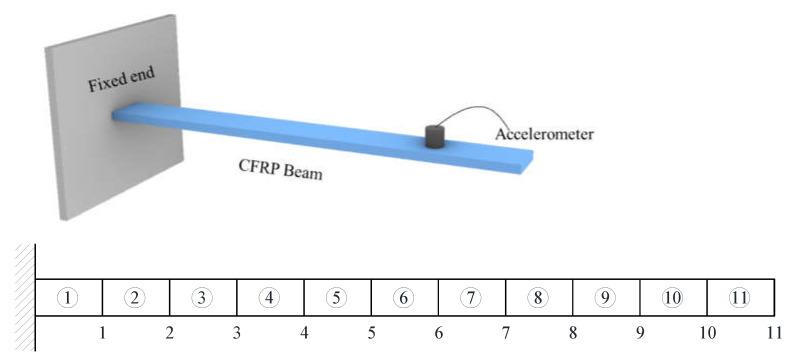
CFRP cantilever beam.

The proposed method is verified again by using the experimental data of a CFRP composite cantilever beam from [39]. The schematic diagram and FEM division diagram of the structure are shown in Figure 6. The three-dimensional size of the structure is 200 mm×20.253 mm×1.7 mm. The basic physical parameters of the material are: elastic modulus $E=1.33\times 10^{5}$ MPa and density $\rho =1.376\times 10^{3}$ kg/m^3^. The structure is evenly divided into 11 beam elements, and the complete FEM has 66 degrees of freedom. From dynamic testing, the first four natural frequencies of the structure in the undamaged and damaged states are measured as shown in Table 4.

**Figure 7:**
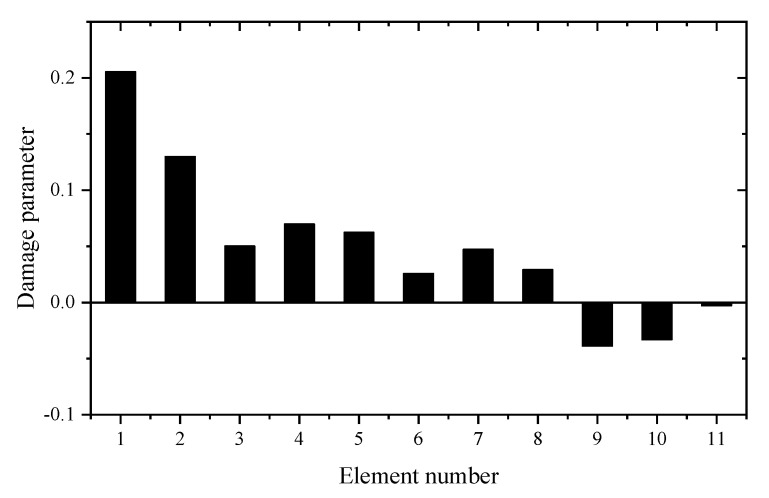
The calculation results of the experimental beam without feedback (element 1 is damaged).

**Figure 8:**
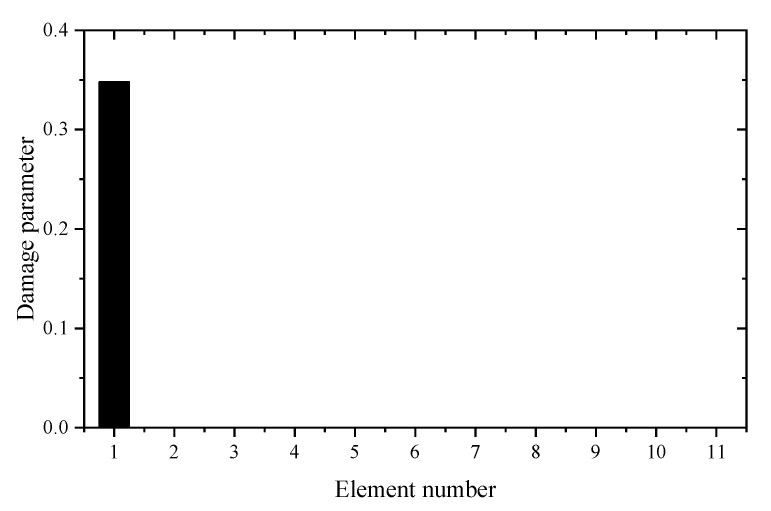
The calculation results of the experimental beam after feedback (element 1 is damaged).

**Figure 9:**
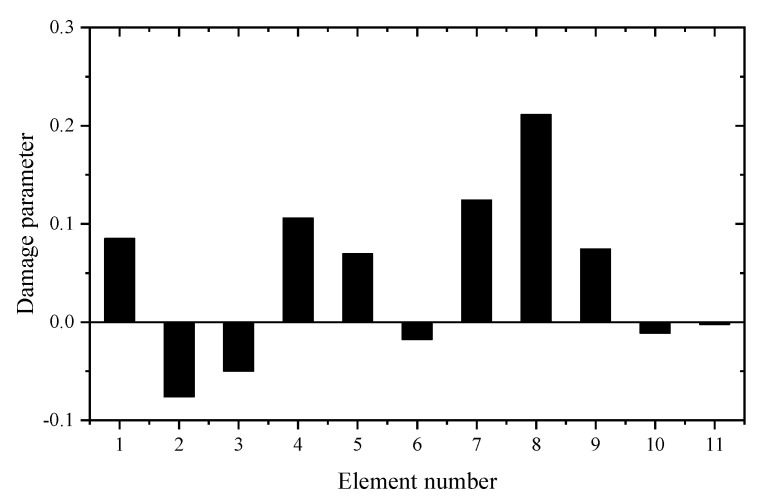
The calculation results of the experimental beam without feedback (elements 5 and 8 are damaged).

**Figure 10:**
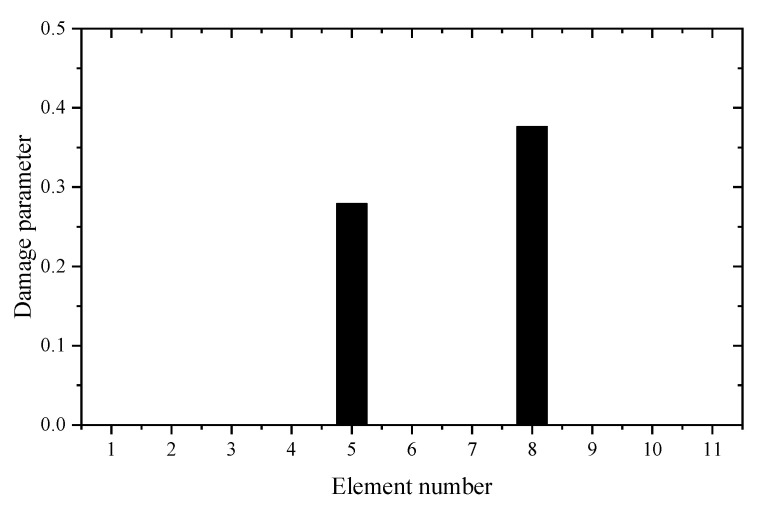
The calculation results of the experimental beam after feedback (elements 5 and 8 are damaged).

The eleven vertical DOFs of nodes 1-11 are chosen as the master DOFs for constructing the reduced FEM. Since only the first four vibration frequencies are measured in modal testing, the damage identification equation is established only by the eigenvalue sensitivities using the reduced FEM. Table 5 gives the sum of squares of the first seven eigenvalues calculated by the reduced FEM with different z. It can be seen from Table 5 that the $\Theta_{z}$ with *z* = 2 is the smallest. This means that the reduced model with *z* = 2 can be used in the following sensitivity calculation. Table 6 shows the first four eigenvalue sensitivities obtained by the complete and reduced model. It can be seen from Table 6 that the calculation results obtained by the reduced model are very close to those obtained by the complete model. It was found again that the proposed sensitivity calculation formula based on reduced FEM has high accuracy. In the case where element 1 is damaged, the first calculation result using the measured frequency data before and after damage is given in Figure 7. It can be found from Figure 7 that unit 1 is the most likely damage unit position. Subsequently, Figure 8 presents the final calculation result based on the feedback. It is clear that Figure 8 indicates that unit 1 is the only damage unit, and the calculated damage parameter is very close to the real damage extent (0.3). In the case where elements 5 and 8 are damaged, the first calculation result using the generalized inverse is given in Figure 9. Using the feedback process, Figure 10 presents the final calculation results. It is clear that Figure 10 indicates that elements 5 and 8 are the true damage units, and the calculated damage parameters are close to the real damage extents (0.2 and 0.3). This shows that the calculation accuracy after feedback is obviously improved.

## 6. Conclusions

In this work, a fast sensitivity analysis technique is firstly proposed by using the reduced FEM, which can effectively solve the mismatch problem between the number of complete DOFs and that of measured DOFs in practice. It was found that the sensitivity calculation values obtained by the reduced FEM are almost the same as those obtained by the complete FEM for low-order vibration modes. Subsequently, the linear equations of structural damage identification are established by using the proposed sensitivity analysis method with the reduced FEM. A feedback-generalized inverse algorithm is then proposed for solving the damage identification equation more accurately. It was found that the ill-conditioned problem of the linear equations can be overcome to a certain extent and more accurate damage assessment results can be obtained. Numerical and experimental examples verified the feasibility of the proposed method. The proposed method may be a very promising tool for sensitivity analysis and damage identification based on reduced FEM. It is worth noting that the proposed method is based on the assumption of linear elastic vibration. It is also assumed that structural damage only causes the change in stiffness matrix and does not cause the change in mass matrix. Sensitivity analysis of nonlinear vibration and corresponding damage identification methods can be further studied in the future.

## Figures and Tables

**Table 1:** The first seven eigenvalues calculated by the complete model and IRS-z reduced models.

Eigenvalue	Complete Model	Reduced Models with Different *z*					
		*z* = 0	*z* = 1	*z* = 2	*z* = 3	*z* = 4	*z* = 5
$\lambda_{1}$	179.83	179.84	179.83	179.83	179.83	179.83	179.83
$\lambda_{2}$	2875.08	2876.83	2875.08	2875.08	2875.08	2875.08	2875.08
$\lambda_{3}$	14,535.90	14,589.09	14,535.90	14,535.90	14,535.90	14,535.90	14,535.90
$\lambda_{4}$	45,855.47	46,520.14	45,855.56	45,855.59	45,855.60	45,855.60	45,855.60
$\lambda_{5}$	111,683.55	116,801.57	111,692.35	111,689.24	111,691.53	111,691.74	111,691.76
$\lambda_{6}$	230,903.67	258,679.47	231,357.32	230,940.61	231,043.55	231,078.20	231,086.91
$\lambda_{7}$	426,272.21	519,557.46	439,504.72	428,184.22	426,440.02	426,405.70	426,614.57
$\sum \lambda_{i}$	832,305.70	959,204.40	846,000.76	834,260.46	832,621.51	832,622.05	832,839.65

**Table 2:** The eigenvalue sensitivities calculated by the complete model and IRS-3 reduced.

Element Number		*i* = 1	*i* = 2	*i* = 3	*i* = 4	*i* = 5	*i* = 6	*i* = 7	*i* = 8
Complete model	$\frac{\partial \lambda_{1}}{\partial \alpha_{i}}$	0.04	0.25	0.67	1.28	2.06	2.97	3.99	5.07
	$\frac{\partial \lambda_{2}}{\partial \alpha_{i}}$	2.30	15.63	40.26	72.44	107.27	139.44	164.07	177.40
	$\frac{\partial \lambda_{3}}{\partial \alpha_{i}}$	25.94	170.42	410.54	665.40	849.09	899.71	800.20	584.09
	$\frac{\partial \lambda_{4}}{\partial \alpha_{i}}$	143.98	900.18	1968.36	2723.06	2722.27	1966.40	898.11	143.05
	$\frac{\partial \lambda_{5}}{\partial \alpha_{i}}$	540.66	3168.82	6082.68	6693.60	4458.42	1362.95	157.58	1913.14
	$\frac{\partial \lambda_{6}}{\partial \alpha_{i}}$	1583.07	8565.07	13,884.80	10,978.45	3430.56	554.32	5897.69	12,864.13
	$\frac{\partial \lambda_{7}}{\partial \alpha_{i}}$	3898.74	19,161.32	25,043.16	12,080.38	1119.43	9787.93	24,129.97	21,063.41
*z* = 3 Reduced model	$\frac{\partial \lambda_{1}}{\partial \alpha_{i}}$	0.04	0.25	0.67	1.28	2.06	2.97	3.99	5.07
	$\frac{\partial \lambda_{2}}{\partial \alpha_{i}}$	2.3	15.64	40.29	72.49	107.35	139.56	164.2	177.53
	$\frac{\partial \lambda_{3}}{\partial \alpha_{i}}$	26.01	170.88	411.48	666.47	850.6	901.9	802.19	585.18
	$\frac{\partial \lambda_{4}}{\partial \alpha_{i}}$	145.42	908.03	1980.33	2726.38	2725.56	1978.32	905.92	144.47
	$\frac{\partial \lambda_{5}}{\partial \alpha_{i}}$	560.17	3268.47	6209.17	6680.96	4407.23	1371.95	155.38	1875.25
	$\frac{\partial \lambda_{6}}{\partial \alpha_{i}}$	1737.96	9337.38	14,838.75	11,067.63	3193.18	603.05	6129.45	12,565.21
	$\frac{\partial \lambda_{7}}{\partial \alpha_{i}}$	3652.25	18,172.58	24,224.74	11,855.25	1054.05	9043.17	23,781.76	21,042.1

**Table 3:** The eigenvector sensitivities calculated by the complete model and IRS-3 reduced ($\times 10^{-2}$).

Element Number		*i* = 1	*i* = 2	*i* = 3	*i* = 4	*i* = 5	*i* = 6	*i* = 7	*i* = 8
Complete model	$\frac{\partial \phi_{1}}{\partial \alpha_{i}}$	0.036	0.254	0.686	1.326	1.786	1.856	1.823	1.697
	$\frac{\partial \phi_{2}}{\partial \alpha_{i}}$	0.018	0.127	0.344	0.670	1.106	1.650	2.303	3.061
	$\frac{\partial \phi_{3}}{\partial \alpha_{i}}$	0.003	0.021	0.059	0.124	0.225	0.372	0.577	0.851
	$\frac{\partial \phi_{4}}{\partial \alpha_{i}}$	−0.008	−0.053	−0.139	−0.256	−0.389	−0.521	−0.632	−0.701
	$\frac{\partial \phi_{5}}{\partial \alpha_{i}}$	−0.013	−0.088	−0.234	−0.438	−0.686	−0.957	−1.230	−1.480
	$\frac{\partial \phi_{6}}{\partial \alpha_{i}}$	−0.012	−0.085	−0.225	−0.425	−0.670	−0.944	−1.228	−1.501
	$\frac{\partial \phi_{7}}{\partial \alpha_{i}}$	−0.007	−0.051	−0.135	−0.256	−0.404	−0.571	−0.746	−0.918
*z* = 3 Reduced model	$\frac{\partial \phi_{1}}{\partial \alpha_{i}}$	0.036	0.254	0.686	1.326	1.786	1.856	1.823	1.697
	$\frac{\partial \phi_{2}}{\partial \alpha_{i}}$	0.018	0.127	0.344	0.670	1.106	1.650	2.303	3.061
	$\frac{\partial \phi_{3}}{\partial \alpha_{i}}$	0.003	0.021	0.059	0.124	0.225	0.372	0.577	0.851
	$\frac{\partial \phi_{4}}{\partial \alpha_{i}}$	−0.008	−0.053	−0.139	−0.256	−0.389	−0.521	−0.632	−0.701
	$\frac{\partial \phi_{5}}{\partial \alpha_{i}}$	−0.013	−0.088	−0.234	−0.438	−0.686	−0.957	−1.230	−1.480
	$\frac{\partial \phi_{6}}{\partial \alpha_{i}}$	−0.012	−0.085	−0.225	−0.425	−0.670	−0.944	−1.228	−1.501
	$\frac{\partial \phi_{7}}{\partial \alpha_{i}}$	−0.007	−0.051	−0.135	−0.256	−0.404	−0.571	−0.746	−0.918

**Table 4:** Natural frequencies obtained by FEM and dynamic testing.

Vibration Mode	1	2	3	4
Analytical values by the undamaged FEM	67.50	422.95	1184.28	2321.37
Experimental values of the undamaged structure	67.49	423.00	1184.53	2321.23
Experimental values when element 1 has 30% stiffness reduction (in [39])	63.28	405.60	1150.23	2271.28
Experimental values when elements 5 and 8 have 20% and 30% stiffness reductions (in [39])	66.75	406.69	1128.32	2281.90

**Table 5:** Eigenvalues of the complete model and reduced model for the experimental beam (×10^6^).

Eigenvalue	Complete Model	Reduced Model					
		*z* = 0	*z* = 1	*z* = 2	*z* = 3	*z* = 4	*z* = 5
$\lambda_{1}$	0.18	0.18	0.18	0.18	0.18	0.18	0.18
$\lambda_{2}$	7.06	7.06	7.06	7.06	7.06	7.06	7.06
$\lambda_{3}$	55.37	55.38	55.37	55.37	55.37	55.37	55.37
$\lambda_{4}$	212.74	212.92	212.74	212.74	212.74	212.74	212.74
$\lambda_{5}$	582.31	583.96	582.31	582.31	582.31	582.31	582.31
$\lambda_{6}$	1303.90	1314.30	1303.90	1303.90	1303.90	1303.90	1303.90
$\lambda_{7}$	2558.40	2608.20	2558.50	2558.40	2558.40	2558.40	2558.40
$\sum \lambda_{i}$	4719.96	4782.00	4720.06	4719.96	4719.96	4719.96	4719.96

**Table 6:** Eigenvalue sensitivities of the complete model and reduced model with *z* = 2 for the experimental beam (×10^6^).

Element Number		*I* = 1	*I* = 2	*I* = 3	*I* = 4	*I* = 5	*I* = 6	*I* = 7	*I* = 8
Complete model	$\frac{\partial \lambda_{1}}{\partial \alpha_{i}}$	0.06	0.04	0.03	0.02	0.01	0.01	0.00	0.00
	$\frac{\partial \lambda_{2}}{\partial \alpha_{i}}$	1.61	0.36	0.03	0.37	0.94	1.29	1.22	0.81
	$\frac{\partial \lambda_{3}}{\partial \alpha_{i}}$	9.22	0.61	5.12	8.27	3.91	0.42	4.86	10.73
	$\frac{\partial \lambda_{4}}{\partial \alpha_{i}}$	26.02	10.92	30.61	7.91	12.81	35.61	12.36	9.49
*z* = 2 Reduced model	$\frac{\partial \lambda_{1}}{\partial \alpha_{i}}$	0.06	0.04	0.03	0.02	0.01	0.01	0.00	0.00
	$\frac{\partial \lambda_{2}}{\partial \alpha_{i}}$	1.61	0.36	0.03	0.37	0.94	1.29	1.22	0.81
	$\frac{\partial \lambda_{3}}{\partial \alpha_{i}}$	9.22	0.61	5.12	8.27	3.91	0.42	4.86	10.72
	$\frac{\partial \lambda_{4}}{\partial \alpha_{i}}$	25.98	10.90	30.57	7.90	12.79	35.56	12.34	9.48

## Abbreviations

FEM	Finite element model
DOF	Degree of freedom
IRS	Improved reduced system
IRS-z	The z-order improved reduced system
GI	Generalized inverse
FGI	Feedback generalized inverse

## References

[1] Huynh T.C., Park J.H., Kim J.T. (2016). Structural identification of cable-stayed bridge under back-to-back typhoons by wireless vibration monitoring. Measurement.

[2] Sharma G.S., Faverjon B., Dureisseix D., Skvortsov A., MacGillivray I., Audoly C., Kessissoglou N. (2020). Acoustic Performance of a Periodically Voided Viscoelastic Medium With Uncertainty in Design Parameters. J. Vib. Acoust..

[3] Liu H.F., Li Z.G. (2019). An improved generalized flexibility matrix approach for structural damage detection. Inverse Probl. Sci. Eng..

[4] Yang Q.W. (2011). A new damage identification method based on structural flexibility disassembly. J. Vib. Control..

[5] Lu Z.R., Zhou J.X., Wang L. (2019). On choice and effect of weight matrix for response sensitivity-based damage identification with model and measurement errors. Mech. Syst. Signal Process..

[6] Krome F., Gravenkamp H. (2016). Analyzing modal behavior of guided waves using high order eigenvalue derivatives. Ultrasonics.

[7] Vieira M.V.C. (2016). Derivatives of eigenvalues and Jordan frames. Numer. Algebra Control. Optim..

[8] Araujo J.M., Dorea C.E.T., Goncalves L., Carvalho J.B., Datta B.N. (2018). Robustness of the Quadratic Partial Eigenvalue Assignment using Spectrum Sensitivities for State and Derivative Feedback Designs. J. Low Freq. Noise Vib. Act. Control..

[9] Emerson B., Lieuwen TJuniper M.P. (2016). Local stability analysis and eigenvalue sensitivity of reacting bluff-body wakes. J. Fluid Mech..

[10] Mousavi M., Holloway D., Olivier J.C. (2020). A new signal reconstruction for damage detection on a simply supported beam subjected to a moving mass. J. Civ. Struct. Heal. Monit..

[11] Teixeira J.S., Stutz L.T., Knupp D.C., Neto A.S. (2020). A new adaptive approach of the Metropolis-Hastings algorithm applied to structural damage identification using time domain data. Appl. Math. Model..

[12] Fox R.L., Kapoor M.P. (1968). Rates of change of eigenvalues and eigenvectors. AIAA J..

[13] Nelson R.B. (1976). Simplified calculation of eigenvector derivatives. AIAA J..

[14] Lim K.B., Junkins J.L., Wang B.P. (1987). Re-examination of eigenvector derivatives. J. Guid. Control. Dyn..

[15] Zhang O., Zerva A. (1996). Iterative method for calculating derivatives of eigenvectors. AIAA J..

[16] Zhang O., Zerva A. (1997). Accelerated iterative procedure for calculating eigenvector derivatives. AIAA J..

[17] Balmes E. Efficient sensitivity analysis based on finite element model reduction. Proceedings of the 16th International Modal Analysis Conference.

[18] Zeng Q.-H. (1995). Highly accurate modal method for calculating eigenvector derivatives in viscous damping systems. AIAA J..

[19] Sondipon A. (2002). Derivative of eigensolutions of nonviscously damped linear systems. AIAA J..

[20] Adhikari S. (2000). Calculation of derivative of complex modes using classical normal modes. Comput. Struct..

[21] Van Der Aa N.P., Ter Morsche H.G., Mattheij R.R. (2007). Computation of eigenvalue and eigenvector derivatives for a general complex-valued eigensystem. Electron. J. Linear Algebra.

[22] Lin R.M., Lim M.K. (1995). Structural sensitivity analysis via reduced-order analytical model. Comput. Methods Appl. Mech. Eng..

[23] Lin R.M., Wang Z., Lim M.K. (1996). A practical algorithm for the efficient computation of eigenvector sensitivities. Comput. Methods Appl. Mech. Eng..

[24] Adhikari S., Friswell M.I. (2006). Calculation of eigensolution derivatives for nonviscously damped systems. AIAA J..

[25] Wu B., Xu Z., Li Z. (2007). Improved Nelson’s Method for computing eigenvector derivatives with distinct and repeated eigenvalues. AIAA J..

[26] Guedria N., Chouchane M., Smaoui H. (2007). Second-order eigensensitivity analysis of asymmetric damped systems using Nelson’s method. J. Sound Vib..

[27] Wang P., Dai H. (2015). Calculation of eigenpair derivatives for asymmetric damped systems with distinct and repeated eigenvalues. Int. J. Numer. Methods Eng..

[28] Wang P., Dai H. (2016). Eigensensitivity of symmetric damped systems with repeated eigenvalues by generalized inverse. J. Eng. Math..

[29] Ruiz D., Bellido J., Donoso A. (2017). Eigenvector sensitivity when tracking modes with repeated eigenvalues. Comput. Methods Appl. Mech. Eng..

[30] Lin R., Ng T. (2019). Eigenvalue and eigenvector derivatives of fractional vibration systems. Mech. Syst. Signal Process..

[31] Yang Q.W., Peng X. (2020). An exact method for calculating the eigenvector sensitivities. Appl. Sci..

[32] Guyan R.J. (1965). Reduction of stiffness and mass matrices. AIAA J..

[33] Irons B. (1965). Structural eigenvalue problems-elimination of unwanted variables. AIAA J..

[34] O’Callahan J. A procedure for an improved reduced system (IRS) model. Proceedings of the 7th International Modal Analysis Conference.

[35] Yang Q., Zhang J., Li C. (2020). An improved IRS method for structure model condensation. Mech. Eng..

[36] Xia Y., Chen T., Shan J. (2014). A novel iterative method for computing generalized inverse. Neural Comput..

[37] Bouhamidi A., Jbilou K., Reichel L., Sadok H. (2011). An extrapolated TSVD method for linear discrete ill-posed problems with Kronecker structure. Linear Algebra Appl..

[38] Hansen P.C. (1992). Analysis of discrete ill-posed problems by means of the L-curve. SIAM Rev..

[39] Behtani A., Tiachacht S., Khatir S., Slimani M., Mansouri L., Bouazzouni A., Waheb M.A. (2020). The sensitivity of modal strain energy for damage localization in composite stratified beam structures. Proceedings of the 13th International Conference on Damage Assessment of Structures.

